# The Disposal of Placenta among Indigenous Groups Globally: An Integrative Literature Review

**DOI:** 10.1155/2023/6676809

**Published:** 2023-10-26

**Authors:** Cecilia Moeti, Fhumulani Mavis Mulaudzi, Molatelo Melitah Rasweswe

**Affiliations:** ^1^Faculty of Healthcare Sciences, Department of Nursing, University of Pretoria, South Africa; ^2^Faculty of Healthcare Science, Department of Nursing, University of Limpopo, South Africa

## Abstract

**Introduction:**

The placenta, or afterbirth, plays a vital role in supplying nutrients and oxygen via the umbilical cord. Western medicine sees the placenta as a medical waste and discards it after delivery. Meanwhile, indigenous groups observe rituals or ceremonies prior to their disposal since it bears sacred importance.

**Aim:**

The aim of the literature review is to review the current literature on indigenous methods of disposing placenta.

**Methods:**

Through the EBSCOhost search engine, the authors had access to the following databases: CINAHL; MEDLINE; E-Journals; Health Sources: Nursing/Academic Edition; Scopus; and African Journals Online. A manual search of the grey literature through Google Scholar and Google Search engines, as well as citation searching using reference lists, was also used. The following keyword searches came up: placental disposal, placental waste, placental release, indigenous placental disposal, traditional placental disposal, cultural placenta, and placental rituals. The authors followed the inclusion criteria of qualitative, quantitative, or mixed research articles or reports from experts and different organisations published between 2013 and 2022 in English. *Findings*. The following three themes with subthemes emerged in the context of this review paper: (1) placental consumption (increases milk production, prevents postpartum depression, and prevents postpartum bleeding); (2) placental burial (burial site determines the child's fate, protection of the child, and fertility); and 3). artifacts (memorabilia).

**Conclusion:**

Indigenous placental disposal methods have a significant value to Indigenous women globally. The rituals performed have a special meaning attached to them. It is important for Western medicine to respect and support indigenous placental disposal methods and ensure safe handling from the healthcare facilities to their homes.

## 1. Introduction

The placenta, also known as “afterbirth,” is an organ that connects the pregnant mother to the fetus (unborn baby) through the umbilical cord [1–3], and it is born immediately after the baby. In addition to being a biological occurrence, childbirth is also a socially and culturally produced event with corresponding symbols and rituals that reflect the social identities and cultural values of each community. In many indigenous cultures, the birth of a baby is not resolved until after the proper disposal of the placenta. Globally, different cultures honour, appreciate, and dispose the placenta differently postdelivery, depending on their traditional meanings, values, beliefs, and practices that are passed from generation to generation [2–4]. Some cultures dispose of it with medical waste, while others show some respect to the placenta, or “afterbirth,” and perform sacred rituals and ceremonies prior to disposal. The beliefs and practices from different traditions are that if the placenta is not disposed properly, it may affect the woman's future fertility prospects or that of her descendants. In those situations, rituals are performed to conform to the belief systems.

The rituals and ceremonies are performed to ensure the safe arrival of the mother and the newborn at the next stage of life. This belief system is common among many traditions globally. For example, Australian home-birthing women use the placenta in various rituals and ceremonies to spiritualise an aspect of birth that is usually overlooked [5]. In Kenya, the Marakwet community believes that proper traditional management and disposal of the placenta contribute to the well-being of both the mother and the baby, as asserted in [3]. In some cultures, the gender of the child determines the placental burial/disposal spot and direction. For instance, in the Marakwet community and Kenyan Luo cosmology, the male and female child's placenta is buried in the right and left-hand direction from the house of delivery, respectively [3, 6].

On the other hand, despite the different beliefs attached to the disposal of the placenta, there are different types of indigenous ways of disposing of the placenta, or “afterbirth”, depending on how it is believed that the mother or a newborn will benefit. These benefits are highly associated with cultural meanings, values, and beliefs that are sacred to the holders. Most of the benefits have a link to the future health predictions of the mother and/or a newborn, while others extend to societal wealth. Some cultures burn it, some bury it [7], and some consume it [8]. In addition to that, some cultures throw it in the bush after the rituals [2]; some dry it and store it for a symbolic reason. Culturally, they believe that these practices may release some of the anxiety that often accompanies pregnancy, labor, birth, and new motherhood [5]. It is, therefore, important for healthcare providers to understand these dynamics of placenta disposal to allow women to practice their beliefs freely.

The aim of this paper is to report on the integrated literature review that focused on the indigenous methods of disposing placentas to enable midwives to have knowledge and understanding of beliefs and practices used to dispose of placentas.

## 2. Design and Method

This review paper adopted an integrative literature review method, which intends to integrate existing empirical and theoretical literature as a way of generating new knowledge. The literature review method followed [9] five stages: stage 1: problem identification; stage 2: literature search; stage 3: data evaluation; stage 4: data analysis; and stage 5: presentation.

## 3. Problem Identification

The initial phase of conducting an integrative literature review is to identify the problem and objective of the review [9]. Despite the availability of legislation allowing women to take home their placentas, there is currently limited literature on strategies to incorporate indigenous placental disposal methods into the health system. This may lead to cross-infections due to improper handling and disposal of the placenta. In this regard, the review question resulting from the identified problem is as follows: what are the indigenous methods of disposing of the placenta?

## 4. Literature Search

The author performed a literature search according to the following inclusion criteria: qualitative, quantitative, or mixed research articles or reports from experts and different organisations or institutions with a vast interest in the indigenous methods of placental disposal. The literature published in English between 2013 and 2022 was included, with the exclusion of letters and commentaries. The University of Pretoria librarian assisted with search strategies such as MeSH (Medical Subject Headings) to increase the robustness of the search.

The search yielded the following search terms: placental disposal, placental waste, placental release, indigenous placental disposal, traditional placental disposal, cultural placenta, and placental rituals. The following databases were investigated using the EBSCOhost search engine: Cumulative Index for Nursing and Allied Health Literature (CINAHL), Medical Literature Analysis and Retrieval System Online (MEDLINE), E-Journals, Health Sources: Nursing/Academic Edition, Scopus, and African Journals Online. Subsequently, a manual search of grey literature (unpublished publications) was undertaken using Google Scholar and Google Search engines, as well as citation searching using reference lists. The author used Rayyan online software to peruse records of relevance by titles and/or abstracts and with adherence to the inclusion and exclusion criteria. Full texts for potentially relevant literature were retrieved, and full texts and/or abstracts were examined and selected based on the inclusion and exclusion criteria.

With the assistance of the university's librarian, the search through electronic databases produced 280 records, while an additional 18 papers were found through grey literature, citation tracking, reference chaining, website searches, etc. 235 records were removed before screening (duplicates: 34; irrelevant to the research question: 89; other reasons: 112). 63 records were screened, and 21 records were excluded. Records sought for retrieval and assessed for eligibility were 42, and after critical appraisal, a further 21 records were excluded (11: irrelevant outcome; 02: a combination of two study subjects; 08: limited rigour). Only 21 records were included in the review (refer to Figure 1 in the supplementary paper for the PRISMA (16) flow diagram of the literature review). The researcher perused, read, and understood the full texts of all 21 records.

### 4.1. Data Evaluation

Following a review of the literature, a selection and perusal of twelve publications was finalised. They were then arranged in a tabular form using the [9] six criteria (the author(s), year of publication, country, design and method, population and sampling, and purpose) on a three-point scale as “high,” “low,” or “not reported” (refer to Table 1). While eight studies employed qualitative techniques, one was a review paper, and three empirical papers adopted a quantitative strategy and used interviews and surveys to gather data.

### 4.2. Data Analysis

Thematic analysis was utilised to analyse the selected publications independently. The paper highlighted, summarized, and identified key findings or meanings relating to the research goal. The key results or meanings were organised into themes and subthemes. The researcher finalised the themes and subthemes for describing indigenous placental disposal systems. The following are the emerging topics and subthemes: (1) theme: placental consumption; subthemes: increases milk production, prevents postpartum depression, and prevents postpartum bleeding. (2) Theme: placental burial; subthemes: burial site determines the child's fate, protection of the child, and fertility. (3) Theme: artifacts and memorabilia.

### 4.3. Presentation

To provide integrative literature reviews, the use of tables is acceptable as claimed in [9]. The information from the primary sources needs to be clear, with evidence to back it up in an orderly sequence of reasoning. The authors presented a table of the results that listed the themes and subthemes and discussed the results and research limitations, with the adherence to [9].

## 5. Results and Discussion

Three themes with subthemes emerged from the examined pieces of literature, as illustrated in Table 2. Discussion of the literature review results occurred simultaneously with the presentation of the themes and subthemes.

### 5.1. Theme 1: Consumption of the Placenta (Placentophagy)

Literature has revealed that the placenta can reportedly be consumed in a variety of ways, including raw, boiled, dried, ground into a powder, and capsuled [5, 13, 15, 22]. The authors further stated that it can be dried, chopped up, and included in a smoothie. The Chinese used it as a remedy for a variety of diseases by drying and powdering it, mixing it with milk, and warming it in the sunlight [19]. Placentophagy was reported among other methods as commonly used by most cultures who cited reduction of postheamorrhage, postnatal depression, and increased breast milk as the benefits of placentophagy [12]. Part of this finding is congruent with [1] who mentioned that almost 3^rd^ of the mothers in the United States of America consume their own placentas, and more than 70% claim that it prevents postpartum depression. However, there was no scientific evidence to support this.

The form of placentophagy most often practiced is “encapsulation,” a process where the placenta is dehydrated, crushed, and packed into capsules [13, 20]. This is consistent with [8, 15] in their study results. The latter further states that 15% of women confirmed to have eaten their placentas raw and between 70 and 80% consumed theirs encapsulated. It has been noticed that placental encapsulation searches continued to rise from the past 10 years [23]. In 2016, a note of caution indicating a well-publicized case was described in which a newborn baby developed a serious *Streptococcus* B infection from a woman who ingested encapsulated placenta, as cited in [3, 17, 20]. The Centres for Disease Control and Prevention, among others, issued a recommendation in response to this event.

#### 5.1.1. Increases Milk Production

Literature papers have identified this subtheme as having an advantageous impact on boosting breast milk supply and, hence, enhancing breastfeeding [8, 13]. Both authors reported that this was advantageous for the mother and her child. Contrary to that, [20] revealed that there was no evidence to support the assertion that placentophagia helps to enhance lactation.

#### 5.1.2. Prevents Postpartum Depression

Consuming the placenta is linked to modifying hormonal imbalance because it is said to lower the risk of postpartum depression and stabilize the mood in [8, 11, 13, 17].

#### 5.1.3. Prevent Postpartum Bleeding

Placentophagy greatly contributes to energy production and the prevention of anemia [22]. The authors further claim that the placenta has a high oxytocin content; hence, they claim that it is useful in preventing postpartum hemorrhage. This is consistent with studies suggesting that the presence of oxytocin hormones in the placenta plays a role in reducing postpartum hemorrhage [13], Field and Janszen in [8]. This outcome is contradicting with [20] study, in which there was no evidence to support the assertion that placentophagy prevents postpartum bleeding.

This review paper has established that researchers claim that there is no scientific rigour to support the benefits of placentophagia. Others, on the other hand, credit benefits to placentophagy, such as improved milk production (which is beneficial to both the mother and the child), mood enhancement, decreased postpartum depression, and exhaustion. The presence of prostaglandin, oestrogen, and iron in the placenta plays a role in the notion. However, [3] indicated that the richness of the latter has no discernible influence on postpartum iron status.

Other studies contend that this method could present certain health risks, such as bacterial or viral infections, or that the presence of trace elements could become dangerous for both the mother and the child. Literature has proven that placentophagy is a common indigenous placental disposal method, yet limited studies have addressed strategies to prevent and control infections that might come with placentophagy.

### 5.2. Theme 2: Placental Burial

The reviewed literature suggested that placenta burial was prevalent in various cultures. The burial place differed from one culture to the next, but the aim was to keep evil spirits, witchcraft, and other practices away from the child. It is indicated that Beninese culture buries the placenta in an unglazed pot which signifies preservation or directly on earth to signify the return to the soil [14]. A tree can be planted on top of the placenta which signifies the child as he/she continues to receive nutrients from the placenta as in the uterus. A specific individual responsible for burying the placenta differs from culture to culture. It is the obligation of an important family member (the father of the child, the grandmother, or a female who has entered the postmenopausal phase) to conduct such a ritual [2, 3].

#### 5.2.1. The Burial Site Determines the Fate of the Child

The customs and beliefs influenced the varying locations of burials. The manner in which the placenta is buried determines what the child becomes later in life [4, 7]. This is inconsistent with the study findings in [10]. Some cultures bury their placentas in a bin or pit in front of their homes, and additionally, they claim that the burial site is influenced by a child's gender [2]. An example is that of the Tonga people who bury the placenta of a boy child on the right side of the house and a girl child on the left [2]. They believe that the girl child may leave the house by virtue of marriage, while the boy child will carry the family name and continue the patriarchal role. This is consistent with the findings of the studies conducted in [3, 24]. The latter revealed that in Marakwet culture, the right hand is symbolic of power and strength, while the left hand is associated with weakness and submission.

The boy child's placental burial takes place beneath the house's central column (the dwelling of the most notable house spirit) while that of a girl child is beneath a bedpost [16]. The Native Hawaiian, Navajo, and Maori tribes believe that by burying the placenta in the homeland, the child is bound to the land and his/her ancestral heritage [3, 15]. The meaning linked to burial also varies by customs and beliefs [22].

#### 5.2.2. Protection of the Child

The placenta is described as a sacred organ that connects the child to the ancestors, spirits, and Mother Earth for protection [2, 4, 7, 20]. In contrast to that, in Nepal, the Lao tribe burns the placenta to prevent spirits and animals from reaching it as they view it as dirt [22]. Some cultures plant a fruit tree on the burial site for the protection of the child and with the belief that the tree will hold major significance in the child's life due to the connection [2]. The burial represents the connection of the child to the soil, spirit, or ancestors [2, 3, 5, 21]. In addition, [18] indicated that the purpose of placental burial was merely for protection. Certain cultures believe that burying symbolizes the life of a baby and the death of a placenta. In this way, when a child dies, he or she will return to earth to reunite with the placenta that protected him or her. The Makwaret pour milk and millet on their way to dispose of the placenta to appease the ancestors so that they can protect the child from any harm [3].

#### 5.2.3. Fertility

In the West African nation of Niger, placenta burial uses a specific method since they believe that it connects a woman's capacity for reproduction. This is consistent with the outcomes from [3, 8], revealing that the placenta restores the woman's fertility by bringing healing to the uterus. Furthermore, the Makwaret traditional birth attendant spreads millet on their way to placenta disposal to prevent the woman from becoming barren. This was because they believe that the placenta predicts a woman's number of children, sexuality, and sexual sequence [3, 8].

In addition to that, the Kikuyu culture symbolises the placenta with fertility; hence, they deposit it in an uncultivated field and place grass and grains on top of it [3, 19]. The Tonga tribe plants the mupundu tree on the placenta burial site. The mupundu tree is known as a fertility tree; hence, they link it with the fertility of a woman [8]. In Hungary, women who desired to stop having children burn their placentas and sprinkle the ashes in their husbands' drinks for them to drink, while in Japanese culture, they believe that eating the placenta would increase a woman's fertility [3].

This review paper has established that the process of placental burial has to be handled and treated with great respect since it carries a special meaning to each culture. The rituals and practices that come with placental burial benefit both the mother and the child. Placental burial also links the child to the soil, earth, spirit, or ancestors. It is a way of introducing the child to the ancestors for protection. The soil or earth also represents fertility; hence, some cultures plant it with a belief of restoring the woman's fertility. Other studies link indigenous placental burial methods with the prevention of hunger, continuity of life, and determination of the child's fate. Other studies link the child to life and the placenta to death; hence, they saw it befitting to bury the placenta [16]. On the same breath, when the child dies, burial takes place where he or she originates (on the soil, where they planted the placenta). The handling of the placenta is common among indigenous groups and is a health hazard.

### 5.3. Theme 3: Artifact

#### 5.3.1. Memorabilia

The literature claimed that a piece of placenta would be dried, painted, and turned into jewellery or poster. These customs are believed to exist because parents wish to remember their offspring and respect the birth of their children [8, 9]. The latter author's studies found that mothers traced the placenta on a sheet of paper, framed and hung in the child's room. The review paper noted that women would create art, pieces of jewellery, etc. to keep as a remembrance and a token of appreciation to the placenta for having kept the child in utero until birth. This method of placental disposal tends to involve a lot of handling and therefore poses a great risk of infection.

## 6. Conclusion

From the above findings, it is eminent that the placenta indigenous groups globally respect the placenta and that its disposal plays a significant value to the indigenous groups globally. It is also clear that different cultures perform certain rituals or practices for disposing of their placentas and have attached a meaning to that. Both the mother and the child enjoy the benefits that come with placental disposal methods. It is in this regard that Western medicine acknowledges respect and supports indigenous placental methods by ensuring the control and prevention of cross-infections.

## 7. Recommendation

Encourage healthcare providers to incorporate indigenous placental disposal methods during antenatal care clinics. Educate indigenous women on the handling of the placenta during transportation from a health care setting to home as well as during disposal at home. Taking into consideration the different indigenous placenta disposal methods emanating from this review paper, healthcare providers should provide information on managing the specifics based on the method used, e.g., the depth of the hole for placental burial and the optimal temperature in respect of the consumption of the placenta. Healthcare providers should also provide cautious information related to the consumption of the placenta to prevent cross-infection.

## Figures and Tables

**Figure 1:**
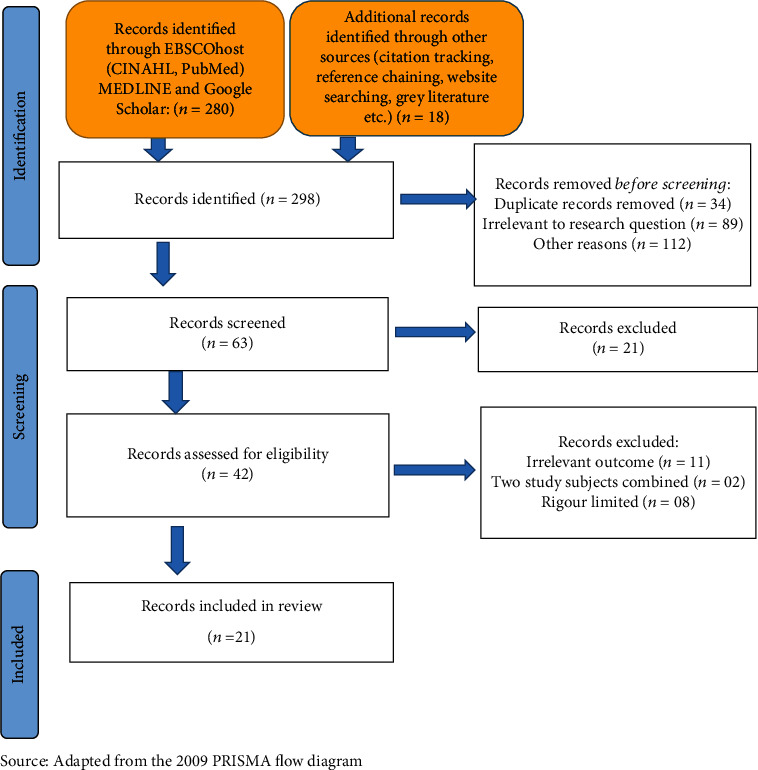
PRISMA flow diagram describing the inclusion process of the integrative literature review.

**Table 1:** Evaluation of publication using Whittemore and Knafl 3-point scale (high, low, and not reported).

Author(s), year, country	Design and method population and sample	Purpose	Quality appraisal (scale: h = high; l = low; nr = not reported)
Adatara et al., 2019 [10], Northern Ghana	A qualitative explorative design Ten (10) out of a total of twenty-seven (27) practicing traditional birth attendants in the study area were purposefully selected from five (5) rural communities in the Bongo District of Ghana for the study. Data were collected through in-depth, unstructured, and individual interviews using a guide.	To explore and describe the role of traditional birth attendants in maternal health care in the rural areas in Ghana	(h) Aims and objectives clearly stated (h) Study design adequately described (h) Research methods appropriate (nr) Explicit theoretical framework (h) Limitations presented (h) Implications discussed

Aziato and Omenyo, 2018 [4], Ghana	Exploratory qualitative design In the purposive sampling technique, both males and females were recruited. To be included in the study, TBAs should have practiced for two years	To gain an in-depth understanding of the initiation of TBAs and their traditional and spiritual practices employed during pregnancy and childbirth in Ghana.	(h) Aims and objectives clearly stated (h) Study design adequately described (h) Research methods appropriate (nr) Explicit theoretical framework (nr) Limitations presented (h) Implications discussed

Benyshek et al., 2018 [11]	Comparison study Data come from the MANA Stats dataset, birth years 2016–2018 This dataset contains complete course of care information for birthing people who planned community births with midwives who participate in the MANA Stats data collection project	To characterize the practice of placentophagy and its attendant neonatal outcomes among a large sample of women in the United States.	(l) Aims and objectives clearly stated (h) Study design adequately described (h) Research methods appropriate (nr) Explicit theoretical framework (h) Limitations presented (h) Implications discussed

Bosco and Diaz, 2018 [12]	Review article	To scientifically document the healthcare team about the risks of placentophagy	(l) Aims and objectives clearly stated (nr) Study design adequately described (nr) Research methods appropriate (nr) Explicit theoretical framework (nr) Limitations presented (l) Implications discussed

Botelle and Willot, 2020 [13], United Kingdom	Qualitative 1752 posts from 956 users across 85 threads from the parenting forums Mumsnet and Netmums were included. A thematic discourse analysis was performed using NVivo.	To explore the discourse produced on two UK Internet forums regarding the practice of placentophagia and how opinions are expressed, discussed, debated, and created through forum participation.	(l) Aims and objectives clearly stated (h) Study design adequately described (h) Research methods appropriate (nr) Explicit theoretical framework (h) Limitations presented (h) Implications discussed

Burns, 2014 [5], Australia	Quantitative explorative 58 Australian home-birthing women, doulas, and independent midwives recruited via web-based forums 43 participants who had recently birthed at home with an independent midwife or were pregnant at the time of the interview and were planning a home birth with an independent midwife	To analyse an alternative childbirth discourse rooted in spirituality, ritual, and sacralisation of space	(h) Aims and objectives clearly stated (l) Study design adequately described (h) Research methods appropriate (nr) Explicit theoretical framework (h) Limitations presented (l) Implications discussed

Chikato and Joseph, 2017 [14], Benin	Commentary article	To draw attention to or present critique on previously published articles	(nr)Aims and objectives clearly stated (nr) Study design adequately described (nr) Research methods appropriate (nr) Explicit theoretical framework (nr) Limitations presented (nr) Implications discussed

Cohen, 2020 [15]	Analytic article	To provide a comprehensive legal analysis of the various uses and modes of consumption of placentas today	(h) Aims and objectives clearly stated (nr) Study design adequately described (l) Research methods appropriate (nr) Explicit theoretical framework (nr) Limitations presented (h) Implications discussed

Corbett et al., 2017 [16], Vietnam	Qualitative study A convenience sample of 8 Hmong women who had recently given birth were interviewed regarding the perinatal experience.	The purpose of this study is to gain an understanding of the meaning of childbirth among Hmong women living in Vietnam	(h) Aims and objectives clearly stated (h) Study design adequately described (h) Research methods appropriate (nr) Explicit theoretical framework (h) Limitations presented (h) Implications discussed

Fadare et al., 2021 [7], Nigeria	Qualitative, descriptive, and exploratory research Semistructured interviews working in the two facilities 22 postpartum mothers and 3 midwives Purposive sampling technique	To assess the perceptions of midwives and mothers on postpartum placenta rituals at selected health centers in Ado-Ekiti, Ekiti State	(h) Aims and objectives clearly stated (h) Study design adequately described (h) Research methods appropriate (h) Explicit theoretical framework (nr) Limitations presented. (h) Implications discussed

Farr et al., 2017 [15]	Review paper		(nr)Aims and objectives clearly stated (nr) Study design adequately described (nr) Research methods appropriate (nr) Explicit theoretical framework (nr) Limitations presented (h) Implications discussed

Johnson et al., 2018 [1]	Review paper	To evaluate the ingestion of processed placenta as a puerperal remedy, the potential risks (trace elements, microorganisms) and possible benefit (hormones in the placental tissue) of such a practice	(h) Aims and objectives clearly stated (h) Study design adequately described (h) Research methods appropriate (nr) Explicit theoretical framework (nr) Limitations presented (h) Implications discussed

Knapp van Bogaert and Ogunbanjo, 2013 [18], South Africa	Ethical and legal review	To discuss legal and ethical considerations regarding postbirth rituals and their relevance to the South African Human Tissue Act	(nr) Aims and objectives clearly stated (nr) Study design adequately described (nr) Research methods appropriate (nr) Explicit theoretical framework (h) Limitations presented (h) Implications discussed

Mainah et al., 2021 [19], Somalia	Explorative, qualitative study 2 married men, 3 TBAs, 2 pregnant mothers, and 2 safe mothers/community midwives. Purposive sampling	To establish the religion-cultural drivers behind placenta disposal; how and where they dispose the placenta and its significance, the manner in which placenta is disposed for the mother, baby, and community at large	(h) Aims and objectives clearly stated (h) Study design adequately described (h) Research methods appropriate (nr) Explicit theoretical framework (nr) Limitations presented (h) Implications discussed

Mota-Rojas et al., 2020 [20]	Review article	The paper is aimed at describing the causes and effects of placentophagia, the endocrine aspects involved, nutritional or analgesic benefits, and the adverse effects that have been published in humans and nonhuman mammals.	(h) Aims and objectives clearly stated (nr) Study design adequately described (nr) Research methods appropriate (nr) Explicit theoretical framework (nr) Limitations presented (l) Implications discussed

Ohaja and Anyim, 2021 [6], Cameroon, Ghana, Kenya, Madagascar, and Mali	This paper is drawing on empirical literature and relevant commentaries	The aim of this paper is to discuss selected rituals and embodied practices surrounding the start of life (pregnancy, birth, and early motherhood).	(h) Aims and objectives clearly stated (nr) Study design adequately described (h) Research methods appropriate (nr) Explicit theoretical framework (nr) Limitations presented (h) Implications discussed

Oyama, 2016 [21], South Africa	Qualitative study Zulu, Black South Africans who were 18 years of age or older from Masxha, KZN Semistructured interviews Purposeful sampling	To gain an understanding of the changes in attitudes towards practicing Zulu rituals during a woman's pregnancy, labor, birth, and postpartum period	(l) Aims and objectives clearly stated (nr) Study design adequately described (h) Research methods appropriate (nr) Explicit theoretical framework (l) Limitations presented (h) Implications discussed

Reed et al., 2019 [2], Australia	A qualitative interpretive approach that was underpinned by a feminist framework 11 women who had expectant management, eight who had active management, and one who was unsure. Semistructured in-depth interviews Purposive sampling	To explore women's experiences of birthing their placenta and, therefore, makes an important contribution to the body of knowledge	(h) Aims and objectives clearly stated (h) Study design adequately described (h) Research methods appropriate (h) Explicit theoretical framework (h) Limitations presented (h) Implications discussed

Rono et al., 2018 [3], Kenya	A descriptive cross-sectional design of qualitative and quantitative methods 186 mothers, selected using multistage cluster sampling, were interviewed: mothers below 20 years of age, another with mothers above 45 years of age, and the third one with married men Purposive sampling	To obtain in part the sociocultural factors that influence the choice of women's birthing site among the Marakwet community of Kenya	(h) Aims and objectives clearly stated (h) Study design adequately described (h) Research methods appropriate (nr) Explicit theoretical framework (nr) Limitations presented (l) Implications discussed

Selander et al., 2013 [8], USA	Quantitative study Internet-based survey on 189 women with an average age of 31 years	To identify a demographic profile of women who have engaged in placentophagia and to evaluate their self-reported motivations for and experiences with the practice	(h) Aims and objectives clearly stated (h) Study design adequately described (h) Research methods appropriate (nr) Explicit theoretical framework (h) Limitations presented (h) Implications discussed

Sharma et al., 2016 [22], Nepal	A qualitative study Five in-depth face-to-face interviews and 14 focus group discussions with mainly women, but also men and health service providers Purposive sampling	To explore social and cultural practices that have health implications in the childbirth and postnatal periods of rural Nepali women	(h) Aims and objectives clearly stated (h) Study design adequately described (h) Research methods appropriate (nr) Explicit theoretical framework (h) Limitations presented (h) Implications discussed

**Table 2:** Presentation of emerging themes and subthemes.

Theme	Subtheme
Consumption of the placenta	Increases milk production Prevents postpartum depression Prevent postpartum bleeding
Placental burial	Fertility
Artifacts	Memorabilia

## Data Availability

Due to the lack of newly generated or analysed data in this review paper, data sharing is not applicable to this publication.
